# Supplementary material for: Spike-timing-dependent ensemble encoding by non-classically responsive cortical neurons
Source: eLife. 2019 Jan 28;8:e42409. doi: 10.7554/eLife.42409 (PMC6391134; doi:10.7554/eLife.42409)
Supplement: Transparent reporting form [file elife-42409-transrepform.docx]

***eLife’s* transparent reporting form**

We encourage authors to provide detailed information *within their submission* to facilitate the interpretation and replication of experiments. Authors can upload supporting documentation to indicate the use of appropriate reporting guidelines for health-related research (see [EQUATOR Network](http://www.equator-network.org/%20)), life science research (see the [BioSharing Information Resource](https://biosharing.org/)), or the [ARRIVE guidelines](http://www.plosbiology.org/article/info:doi/10.1371/journal.pbio.1000412) for reporting work involving animal research. Where applicable, authors should refer to any relevant reporting standards documents in this form.

If you have any questions, please consult our Journal Policies and/or contact us: [editorial@elifesciences.org](mailto:editorial@elifesciences.org).

**Sample-size estimation**

- You should state whether an appropriate sample size was computed when the study was being designed
- You should state the statistical method of sample size computation and any required assumptions
- If no explicit power analysis was used, you should describe how you decided what sample (replicate) size (number) to use

Please outline where this information can be found within the submission (e.g., sections or figure legends), or explain why this information doesn’t apply to your submission:

Sample size information can be found under Methods, Behavior (page 25, lines 530-534).

**Replicates**

- You should report how often each experiment was performed
- You should include a definition of biological versus technical replication
- The data obtained should be provided and sufficient information should be provided to indicate the number of independent biological and/or technical replicates
- If you encountered any outliers, you should describe how these were handled
- Criteria for exclusion/inclusion of data should be clearly stated
- High-throughput sequence data should be uploaded before submission, with a private link for reviewers provided (these are available from both GEO and ArrayExpress)

Please outline where this information can be found within the submission (e.g., sections or figure legends), or explain why this information doesn’t apply to your submission:

The number of animals used in the study are indicated under Methods, Implant preparation and surgery (page 25, lines 537-538). The number of units recorded is indicated in the Results (page 6, lines 126-127).

For technical replicates, we used several procedures to ensure that our results were replicable. 1) All bandwidths for Kernel Density Estimation were set via maximum likelihood 10-fold cross-validation. 2) 10-fold cross-validation was used to determine decoding performance and this process was repeated 124 times with randomly generated folds 3) All decoding performance results were determined to be statistically significant (Mann Whitney U test, two-sided, p <0.05) relative to two controls: i) the decoding performance of spike trains generated by sampling ISIs from the total set of observed ISIs with replacement. ii) the decoding performance when stimulus type and behavioral choice labels were randomly permuted. Both of these controls were repeated 1240 times. All of this information can be found under Methods, ISI-based single-trial Bayesian decoding.

**Statistical reporting**

- Statistical analysis methods should be described and justified
- Raw data should be presented in figures whenever informative to do so (typically when N per group is less than 10)
- For each experiment, you should identify the statistical tests used, exact values of N, definitions of center, methods of multiple test correction, and dispersion and precision measures (e.g., mean, median, SD, SEM, confidence intervals; and, for the major substantive results, a measure of effect size (e.g., Pearson's r, Cohen's d)
- Report exact p-values wherever possible alongside the summary statistics and 95% confidence intervals. These should be reported for all key questions and not only when the p-value is less than 0.05.

Please outline where this information can be found within the submission (e.g., sections or figure legends), or explain why this information doesn’t apply to your submission:

A general description of the statistical analyses used can be found under Methods, Statistical analysis (page 46, lines 994-998). For each statistical test, the statistical methods used are specified as well as the exact p-value. These can be found throughout the Results section and figure legends.

(For large datasets, or papers with a very large number of statistical tests, you may upload a single table file with tests, Ns, etc., with reference to sections in the manuscript.)

**Group allocation**

- Indicate how samples were allocated into experimental groups (in the case of clinical studies, please specify allocation to treatment method); if randomization was used, please also state if restricted randomization was applied
- Indicate if masking was used during group allocation, data collection and/or data analysis

Please outline where this information can be found within the submission (e.g., sections or figure legends), or explain why this information doesn’t apply to your submission:

No randomization was used because all animals underwent the same training protocol and all cells that met criteria were included for analysis.

**Additional data files (“source data”)**

- We encourage you to upload relevant additional data files, such as numerical data that are represented as a graph in a figure, or as a summary table
- Where provided, these should be in the most useful format, and they can be uploaded as “Source data” files linked to a main figure or table
- Include model definition files including the full list of parameters used
- Include code used for data analysis (e.g., R, MatLab)
- Avoid stating that data files are “available upon request”

Please indicate the figures or tables for which source data files have been provided:

The source data for Figures 2A,B and 5B will be uploaded. The model parameters for the recurrent neural network have been fully described in Methods, Recurrent neural network model (pages 41-45). The code used and sample data is available at <https://github.com/badralbanna/Insanally2017> (Methods, page 46, line 1000)
